# A Novel Endogenous Inhibitor of the Secreted Streptococcal NAD-Glycohydrolase

**DOI:** 10.1371/journal.ppat.0010035

**Published:** 2005-12-02

**Authors:** Michael A Meehl, Jerome S Pinkner, Patricia J Anderson, Scott J Hultgren, Michael G Caparon

**Affiliations:** 1 Department of Molecular Microbiology, Washington University School of Medicine, St. Louis, Missouri, United States of America; 2 Department of Medicine, Washington University School of Medicine, St. Louis, Missouri, United States of America; 3 Howard Hughes Medical Institute, Washington University School of Medicine, St. Louis, Missouri, United States of America; University of California at San Diego, United States of America

## Abstract

The *Streptococcus pyogenes* NAD-glycohydrolase (SPN) is a toxic enzyme that is introduced into infected host cells by the cytolysin-mediated translocation pathway. However, how *S. pyogenes* protects itself from the self-toxicity of SPN had been unknown. In this report, we describe immunity factor for SPN (IFS), a novel endogenous inhibitor that is essential for SPN expression. A small protein of 161 amino acids, IFS is localized in the bacterial cytoplasmic compartment. IFS forms a stable complex with SPN at a 1:1 molar ratio and inhibits SPN's NAD-glycohydrolase activity by acting as a competitive inhibitor of its β-NAD^+^ substrate. Mutational studies revealed that the gene for IFS is essential for viability in those *S. pyogenes* strains that express an NAD-glycohydrolase activity. However, numerous strains contain a truncated allele of *ifs* that is linked to an NAD-glycohydrolase−deficient variant allele of *spn*. Of practical concern, IFS allowed the normally toxic SPN to be produced in the heterologous host *Escherichia coli* to facilitate its purification. To our knowledge, IFS is the first molecularly characterized endogenous inhibitor of a bacterial β-NAD^+^−consuming toxin and may contribute protective functions in the streptococci to afford SPN-mediated pathogenesis.

## Synopsis

The gram-positive bacterium *Streptococcus pyogenes* is a human pathogen that causes a wide range of infections from pharyngitis (“strep throat”) to invasive necrotizing fasciitis (“flesh-eating disease”). While strep throat responds to antibiotic therapy, more invasive infections caused by *S. pyogenes* often require surgical intervention. It is currently unknown exactly how the bacteria can switch between the different types of infection, but one possibility is via a mechanism by which the bacterium injects a bacterial protein toxin (*S. pyogenes* NAD-glycohydrolase [SPN]) into human skin cells, causing their death. In this study, the authors have shown that the injected toxin also has the ability to affect the bacteria. A second protein neutralizes SPN to ensure the bacteria are immune to its toxic effects. Consequently, *S. pyogenes* has developed a valuable weapon in its arsenal to promote its survival by ensuring the safe production of SPN, through its own protection by immunity factor for SPN, enabling the delivery of active SPN into human cells. The process reported in this paper may ultimately help create therapeutic inhibitors of SPN and possibly other SPN-like toxins implicated in microbial disease progression.

## Introduction

Bacterial pathogens secrete a multitude of factors that are utilized to advance the infectious process. Many of the secreted factors exhibit an enzymatic activity that is directed against host-specific targets or are activated by host-specific functions. However, a few secreted enzymes are quite promiscuous and have the ability to adversely affect both the microbe and the host cell. Because of this potential self-toxicity, bacteria must develop mechanisms to protect themselves from the deleterious effects of these universally toxic enzymes in order to successfully use them in pathogenesis. One toxic enzyme, the secreted nicotinamide adenine dinucleotide (NAD)–glycohydrolase of *Streptococcus pyogenes* (SPN, also named NGA [1]), has recently been shown to be injected into the host cell cytoplasm via a specialized translocation process known as cytolysin-mediated translocation (CMT) [2,3]. However, how *S. pyogenes* manages the potential self-toxicity of SPN is unknown.

SPN is one of several secreted toxins that are thought to contribute to the pathogenesis of the numerous diseases that *S. pyogenes* can cause. These range from superficial (pharyngitis, impetigo) to life threatening (toxic shock syndrome, necrotizing fasciitis) [4]. The contribution that any one toxin makes to a specific *S. pyogenes* disease is generally not understood. However, SPN has several activities that suggest it may be important for pathogenesis. As an NAD-glycohydrolase, its most well characterized activity is its ability to cleave β-NAD^+^ at the ribose-nicotinamide bond to generate ADP-ribose and the potent vasoactive compound nicotinamide [5−7]. Similar to several other NAD-glycohydrolases, SPN has also been reported to have a cyclase activity capable of converting β-NAD^+^ into cyclic ADP-ribose, a potent second messenger for calcium mobilization [8]. The observation that SPN can transfer ADP-ribose to certain synthetic substrates has suggested that SPN may ADP-ribosylate an important host protein in order to modify the function of that protein [1]. However, the roles that any of these activities may contribute to pathogenesis remains to be established.

Studies using in vitro models of streptococcal pathogenesis have provided evidence that SPN can alter host cell behavior following its translocation into the cytosolic compartment [2,3]. One effect of intracellular SPN is an enhanced cytotoxic response that results in the rapid death of the infected host cell [2,3]. The basis of the cytotoxic response is not understood; however, any of SPN's enzymatic activities could potentially have deleterious effects on host cell viability. For example, if left unchecked, SPN's robust NAD-glycohydrolase activity would likely result in a significant reduction in the intracellular stores of β-NAD^+^. As a co-factor, β-NAD^+^ is universally important in numerous essential redox and energy-producing biological reactions (for a recent review, see [9]). Depletion of β-NAD^+^ would be likely be toxic for both the intoxicated host cell and the streptococcal cell producing SPN. Toxicity for bacterial cells would explain the fact that it has not been possible to clone and express the intact gene for SPN in heterologous hosts such as *Escherichia coli* (M. G. Caparon, unpublished data). Thus, successful production of SPN by its native *S. pyogenes* host likely requires some mechanism for management of its toxic properties.

In this report, we describe immunity factor for SPN (IFS), a novel endogenous inhibitor of the NAD-glycohydrolase activity of SPN. These studies show that the ability of *S. pyogenes* to express and secrete an active SPN has an essential dependence on the production of IFS in its cytoplasmic compartment. Furthermore, co-expression of SPN and IFS allows the normally toxic SPN to be produced at high levels in *E. coli* and allows SPN to be exported as an active enzyme into the periplasmic space. Fractionation of both recombinant and streptococcal extracts identified a cytoplasmic inhibition of NAD-glycohydrolase activity that was dependent on IFS. The mechanism of inhibition involves the ability of IFS to bind tightly to SPN and to act as a competitive inhibitor of its β-NAD^+^ substrate. Taken together, these data reveal how *S. pyogenes* protects itself from the toxic effects of SPN through its expression of IFS, a novel inhibitor of a β-NAD^+^−consuming microbial toxin.

## Results

### Efficient Cloning and Expression of SPN

**Figure 1:**
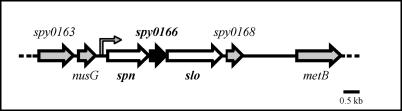
The *spn-slo* Operon Includes the Open Reading Frame *spy0166* The figure depicts the *spn-slo* chromosomal operon from *S. pyogenes* M1 strain SF370 [18]. A promoter upstream of *spn* (bent arrow) drives expression of *spn* and *slo.* Assignment of open reading frames is taken from the annotation of the SF370 genome.

The fact that it has not been possible to clone SPN in any *E. coli* expression vector led us to hypothesize that *S. pyogenes* must have a factor that protects it from any potential toxicity associated with SPN. In searching for this putative factor, we noted that the gene for SPN is present in an operon that includes the gene encoding SLO, the pore-forming component of the CMT injection pathway (Figure 1). Of interest, the operon also includes an open reading frame (*spy0166,* Figure 1) that could potentially encode a small protein (161 amino acids, 18.8 kDa) and would be negatively charged at neutral pH (predicted pI = 5.39). The Spy0166 protein lacks a gram-positive Sec-dependent signal peptide (see Materials and Methods) and would therefore likely serve its role as a soluble cytoplasmic protein since the Sec pathway is the only available route for protein export in *S. pyogenes*. These properties are also characteristic of some protease inhibitors [10,11] and chaperones of proteins translocated by the type III secretion systems of gram-negative bacteria [12], both of which can increase the efficiency of expression of their partner proteins in *E. coli* and in their native hosts [13−16]. Based on these observations, we tested whether *spy0166* from *S. pyogenes* strain JRS4 could support the expression of *spn* in *E. coli* when both genes were placed under the control of the arabinose-inducible promoter on the vector pBAD/gIIIB (see Materials and Methods). In this construct (pMAM3.18), the native signal sequence of SPN was replaced with the gene III signal sequence (for efficient periplasmic targeting) while 6X-HIS and c-myc epitope tags were grafted on the carboxy-terminal end of Spy0166. Unlike any previous SPN expression construct, pMAM3.18 efficiently transformed *E. coli* (>10^4^ transformants/μg DNA) with high fidelity (no nucleotide substitutions, as determined by sequence analysis of several clones). Furthermore, there was no loss of viability following induction of the vector's promoter and fractionation of the resulting cultures revealed SPN in the periplasmic fraction and Spy0166 in the cytoplasmic fraction as detected by Western blot analysis (data not shown).

### Spy0166 Inhibits the Glycohydrolase Activity of SPN

**Figure 2:**
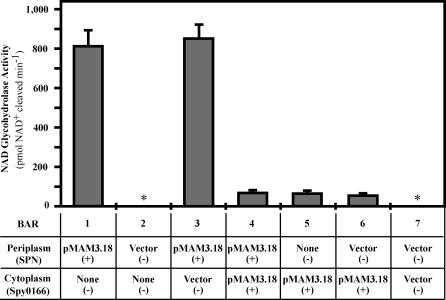
SPN Activity Is Inhibited by IFS (Spy0166) The labeled bars indicate the NAD-glycohydrolase activities of mixtures of isolated periplasm (as a source of SPN) and cytoplasm (as a source of Spy0166) from various *E. coli* strains containing the plasmids indicated. The presence (+) or absence (−) of either SPN or Spy0166 in a particular periplasmic or cytoplasmic extract is indicated. Vector is pBAD/gIIIB containing no inserted streptococcal DNA. Asterisk indicates that the level of NAD-glycohydrolase activity was below the limit of detection of 50 pmol/min. Due to its ability to inhibit NAD-glycohydrolase activity, Spy0166 was renamed IFS. Data represent the mean ± standard error of the mean derived from three independent experiments.

**Figure 3:**
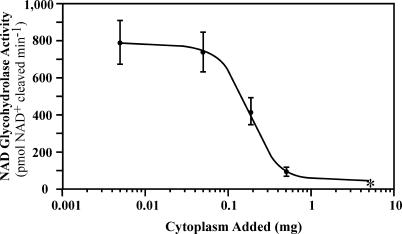
Dose-Dependent Inhibition by IFS NAD-glycohydrolase activity produced by a constant concentration of periplasmic extract (as a source of SPN) mixed with increasing concentrations of a cytoplasmic extract (as a source of IFS), both of which were prepared from TOP10(pMAM3.18). Asterisk indicates that the level of NAD-glycohydrolase activity was below the limit of detection of 50 pmol/min. Data represent the mean ± standard error of the mean derived from three independent experiments.

Since SPN's β-NAD^+^−consuming glycohydrolase activity may have contributed to the protein's apparent toxicity for *E. coli*, it was of interest to determine whether Spy0166 could inhibit SPN's ability to cleave β-NAD^+^. The strategy was to mix together periplasmic and cytoplasmic fractions in various combinations following arabinose induction of *E. coli* strains containing either pMAM3.18 or the vector alone. Upon analysis, the periplasmic fractions derived from pMAM3.18, but not vector-containing strains, demonstrated a robust NAD-glycohydrolase activity (compare bars 1 and 2, Figure 2). Furthermore, the activity produced by pMAM3.18 was not affected by mixing with the cytoplasmic fraction derived from a strain containing the vector alone (bar 3, Figure 2). In contrast, mixing an SPN-containing periplasmic fraction with an Spy0166-containing cytoplasmic fraction resulted in an inhibition of NAD-glycohydrolase activity to near background levels (bar 4, Figure 2), and the degree of inhibition was dependent upon the amount of Spy0166-containing cytoplasmic extract mixed with SPN (Figure 3). In the absence of SPN, Spy1066 had no glycohydrolase activity of its own (bars 5, 6, and 7, Figure 2). Based on its ability to inhibit NAD-glycohydrolase activity and support the viability of SPN-expressing *E. coli,* Spy0166 was renamed IFS.

### Two Alleles of *spn* and *ifs*

**Figure 4:**
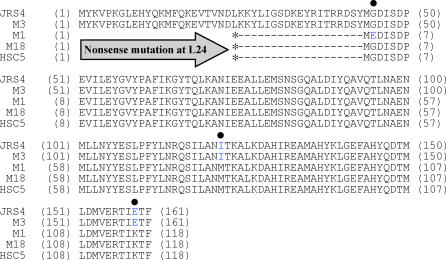
Two Alleles of *ifs* A multiple alignment of *ifs* from several *S. pyogenes* strains is shown. A nonsense mutation in the codon for leucine 24 (indicated by asterisk) produces a truncated *ifs* open reading frame in several strains. The circles above the sequence show the position of several other polymorphic residues. The *ifs* loci were taken from the following genomes: M1 (SF370, *ifs* = Spy0166), M13 (MGAS315, *ifs* = Spy_M30129), M18 (MGAS8232, *ifs* = Spy_M180164), and strains JRS4 and HSC5.

**Figure 5:**
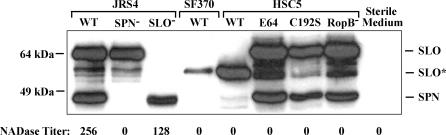
Expression of Enzymatically Active and Inactive SPN Proteins A Western blot analysis of cell-free culture supernatant fluids from various *S. pyogenes* strains is shown. The blot was developed with an antiserum that recognizes the proteins indicated on the left, including SPN, full-length SLO, and a truncated form of SLO (SLO*) generated through a specific cleavage by the streptococcal SpeB cysteine protease [52]. The lanes labeled under JRS4 include JRS4 itself (WT), SPN mutant SPN1 (SPN^−^), and SLO mutant SLO1 (SLO^−^), and under SF370, SF370 itself (WT). Lanes under HSC5 include HSC5 itself (WT), HSC5 grown in the presence of the protease inhibitor E64 (E64), and mutants of HSC5: catalytically deficient SpeB mutant JWR10 (C192S) and protease regulatory mutant MNN100 (RopB^−^). The NAD-glycohydrolase activity titer (NADase titer) of each supernatant fluid is shown at the bottom of each lane. The data shown are representative of results from three independent experiments.

Comparison of *spn* operon sequences among the genomes available for several *S. pyogenes* strains along with sequences obtained from two other commonly studied strains (JRS4 [M6 serotype, 17] and HSC5 [M14 serotype, unpublished data]) revealed the presence of at least two distinct alleles of *ifs*. A distinguishing characteristic is the presence of a nonsense mutation that converts the codon encoding leucine at position 24 (TAT) to a stop codon (TAA) (Figure 4). As a result, the annotation of the genomes of SF370 [18] and MGAS8232 [19] lists the codon for methionine at position 44 as the initiation codon of the IFS open reading frame (Figure 4). The strain used to characterize IFS in the experiments described in the previous section contains the longer allele (JRS4, Figure 4). In addition, it had been previously reported that there are at least two distinct alleles of *spn* that differ by five nonsynonymous substitutions [1]. There was a correlation between which *spn* allele a given strain contained and whether or not it produced immunoreactive SPN [1]. Of the strains analyzed in this study, JRS4 is known to produce active SPN [2] and has the allele associated with active expression (JRS4-WT, Figure 5). Furthermore, the NAD-glycohydrolase activity produced by this strain is completely dependent upon SPN (SPN^−^, Figure 5). In contrast, HSC5 has the allele that was associated with the absence of an ability to produce a detectable SPN protein similar to that reported for M1 strain SF370 [1]. As predicted by this comparison, HSC5 failed to produce any detectable NAD-glycohydrolase activity and any immunoreactive SPN protein (compare HSC5-WT and SF370, Figure 5). One difference between JRS4, SF370, and HSC5 is that the latter two strains are strong producers of the SpeB cysteine protease, while the former is not. Since it has been reported that SpeB can completely degrade SPN [20], HSC5\′s ability to produce SPN was examined in the presence of the cysteine protease inhibitor E64. With this treatment, HSC5 now contained the SPN protein at levels similar to JRS4 (compare JRS4-WT and HSC5-E64, Figure 5). However, the HSC5 SPN still lacked any detectable NAD-glycohydrolase activity (HSC5-E64, Figure 5). This was not a result of E64 treatment or any other inhibitory factor in HSC5 supernatants, because (1) E64 did not inhibit JRS4 SPN (data not shown), (2) elimination of protease activity through mutation of SpeB's active site residue or a deletion of an essential activator of *speB* transcription allowed HSC5 to produce SPN that still exhibited no detectable NAD-glycohydrolase activity (HSC5-C192S and HSC5-RopB^−^, Figure 5), and (3) mixing HSC5 and JSR4 supernatants did not result in inhibition of the JRS4 SPN (data not shown). Consistent with a previous report [20], strain SF370 produced a low amount of SPN in the presence of E64 by Western blot analysis with no detectable NAD-glycohydrolase activity (data not shown). Taken together, these data indicate that in addition to possessing two alleles for *ifs,* the streptococcal population contains at least two major alleles of *spn,* and the product of one of these lacks detectable NAD-glycohydrolase activity.

### Cytoplasmic Extracts of *S. pyogenes* Contain an Allele-Dependent Inhibitory Activity

**Figure 6:**
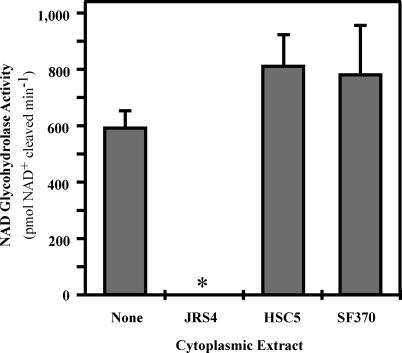
A Cytoplasmic Inhibitory Activity The NAD-glycohydrolase activities of a mixture of JRS4 supernatant (as a source of SPN) and cytoplasmic extracts prepared from the indicated strains are shown. Asterisk indicates that the level of NAD-glycohydrolase activity was below the limit of detection of 50 pmol/min. Data represent the mean ± standard error of the mean derived from three independent experiments.

The data presented above suggest that the function of IFS is to inhibit the NAD-glycohydrolase activity of SPN in the cytoplasmic compartment of *S. pyogenes*. In addition, available data indicate linkage between *spn* and *ifs* alleles. Strains that contain the *spn* allele associated with activity (e.g., JRS4) contain the larger *ifs* open reading frame [see also MGAS315 genome, 21], while those that possess the *spn* allele that does not express activity (e.g., HSC5, SF370) have truncated *ifs* [see also MGAS8232 genome, 19]. To directly test whether *S. pyogenes* contains an inhibitory activity for SPN and the possible influence of the *ifs* allele, the ability of cytoplasmic extracts from various strains to inhibit NAD-glycohydrolase activity was evaluated. Consistent with the behavior of IFS cloned from JRS4 (see above), cytoplasmic extracts of JRS4 contained an activity that inhibited the NAD-glycohydrolase activity of the secreted form of JRS4 SPN (Figure 6). In contrast, cytoplasmic extracts from either HSC5 or SF370 did not contain an activity that inhibited the NAD-glycohydrolase activity of JRS4 SPN (Figure 6). Extracts prepared from HSC5 lacked activity even when prepared in the presence of the protease inhibitor E64 (data not shown).

### Cytoplasmic Inhibitory Activity Was Dependent on IFS

**Figure 7:**
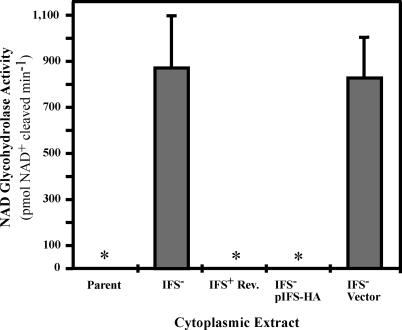
Inhibitory Activity Is Due to IFS The NAD-glycohydrolase activities of a mixture of JRS4 supernatant (as a source of SPN) and cytoplasmic extracts prepared from several derivatives of JRS4 SPN^−^ mutant SPN1 are shown. Lanes include SPN^−^ mutant itself (Parent), a derivative of SPN^−^ mutant with an in-frame deletion in *ifs* (IFS^−^), a sibling of the SPN^−^ IFS^−^ deletion mutant that reverted to wild-type *ifs* (IFS^+^ Rev.), the SPN^−^ IFS^−^ deletion mutant containing a plasmid-encoded HA-tagged version of IFS (IFS^−^ pIFS-HA), and the pABG5 plasmid vector lacking *ifs* (IFS^−^ Vector). Asterisk indicates that the level of NAD-glycohydrolase activity was below the limit of detection of 50 pmol/min. Data represent the mean ± standard error of the mean derived from three independent experiments.

To determine if the inhibitory activity detected in JRS4 was due to IFS, an attempt was made to construct an in-frame deletion in the gene for IFS. The method for mutagenesis proceeds via the generation of a tandem duplication of mutant and wild-type alleles in the genome that can resolve to either allele by recombination. Typically, chromosomes with either allele are isolated at similar frequencies among the progeny [22]. However, while it was possible to produce the merodiploid intermediate strain at the *ifs* locus, all progeny recovered following resolution of the duplication contained a copy of the wild-type allele (30 of 30 tested). In contrast, when mutagenesis was conducted in a JRS4 SPN^−^ mutant, progeny with the mutant or wild-type *ifs* alleles were recovered at equal frequencies (four mutants, four wild-type, eight tested). These data suggest that IFS is essential for viability when SPN is encoded in the genome. Consistent with this, when progeny of the SPN^−^ mutant were analyzed, only those progeny with the wild-type *ifs* allele could inhibit SPN NAD-glycohydrolase activity (compare IFS^+^ Rev. to IFS^−^, Figure 7). Inhibitory activity was restored in the IFS^−^ mutant upon the introduction of an HA-tagged version of the JRS4 *ifs* on a plasmid (compare pIFS-HA to Vector, Figure 7). The presence of IFS in the cytoplasm of the complementing strain was verified by Western blot analysis using antiserum to the HA epitope tag (data not shown). Taken together, these data indicate that in the absence of IFS, expression of SPN is likely lethal for JRS4 and that the inhibitory activity detected in cytoplasmic extracts is dependent on IFS.

### An SPN-IFS Complex

**Figure 8:**
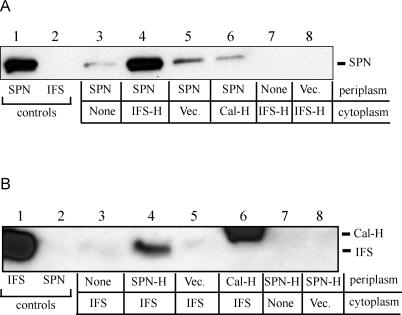
SPN and IFS Form a Complex Ni-NTA pull-down assays detect the formation of a complex between IFS and SPN. (A) Periplasmic extracts (periplasm) from *E. coli* strain TOP10(pMAM3.8) expressing an non−6X-His–tagged version of SPN (SPN), the pBAD/pIIIB vector (Vec.), or buffer (None) were mixed with cytoplasmic extracts (cytoplasm) from TOP10(pMAM3.19) that expressed a 6X-His and c-myc–tagged version of IFS (IFS-H), the pBAD/gIIIB vector (Vec.), a plasmid expressing a 6X-His and c-myc–tagged calmodulin (Cal-H), or buffer (None). Proteins released following pull-down with Ni-NTA agarose were analyzed by Western blot with an antiserum to detect SPN, as shown. (B) Periplasmic extracts from TOP10(pMAM3.18) expressing a strain a 6X-His–tagged version of SPN (SPN-H) were mixed with cytoplasmic extracts prepared from TOP10(pMAM3.21), which expresses IFS with a c-myc epitope, but lacking a 6X-His tag. Other designations are as in (A). Proteins released following pull-down with Ni-NTA agarose were analyzed by Western blot with an antiserum to detect the c-myc tags of IFS and Cal-H, as shown. Controls are purified IFS and SPN that were not subjected to pull-down, and the migration of IFS, SPN, and Cal-H is indicated to the right.

The ability of IFS to inhibit SPN's enzymatic activity suggested that the two proteins interact. To test this, pull-down assays were conducted following mixing periplasmic extracts (as a source of SPN) and cytoplasmic extracts (as a source of IFS) prepared from various *E. coli* strains. In the first assay, the cytoplasmic extract was prepared from a strain expressing IFS with C-terminal 6X-His and c-myc epitope tags (IFS-H) and periplasm was prepared from a strain expressing SPN and IFS. Following mixing, IFS-H was isolated via selective binding to Ni-NTA agarose and any co-isolation of SPN assessed by Western blot analysis. In the absence of IFS-H, SPN demonstrated some affinity for Ni-NTA agarose under these conditions (lane 3, Figure 8A). However, in the presence of IFS-H, SPN was readily co-isolated (lane 4, Figure 8A) at levels substantially greater than background observed with cytoplasmic extracts from strains containing the vector alone (lane 5, Figure 8A) or an irrelevant 6X-His–tagged protein (lane 6, Figure 8A). The reciprocal experiment yielded similar results, where IFS (without a 6X-His tag) was selectively co-isolated only when incubated with periplasm containing 6X-His–tagged SPN (SPN-H) (lane 4, Figure 8B) and not in the absence of any periplasmic extract (lane 3, Figure 8B), the presence of periplasmic extracts prepared from strains expressing the plasmid vector alone (lane 5, Figure 8B), or an irrelevant 6X-His–tagged protein (lane 6, Figure 8B).

### Characteristics of the SPN-IFS Complex

**Figure 9:**
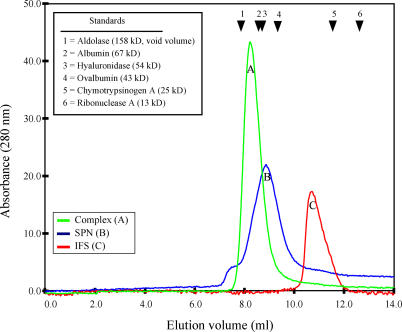
Gel Filtration Chromatography of SPN, IFS, and the SPN-IFS Complex Purified SPN (B), IFS (C), or the SPN-IFS (A) complex was subjected to gel filtration chromatography over Superdex 75 HR 10/30, and their elution profiles are overlaid. Based on the elution of several standards (identities of the standards are shown, and their elution volumes are indicated by the corresponding arrows), the calculated molecular weights of SPN, IFS, and the complex are 52.4 ± 1.5, 31.5 ± 3.2, and 67.6 ± 3.2 kDa, respectively. These calculations were based on the average elution volumes derived from at least three independent column applications.

**Figure 10:**
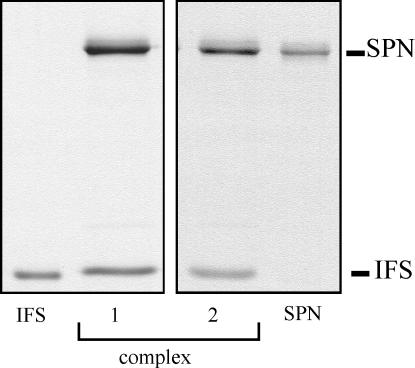
Analysis of the SPN-IFS Complex Several fractions of the high-molecular-weight peak arising from gel filtration chromatography of an SPN-IFS complex were analyzed by SDS-PAGE as shown. Purified SPN and IFS are included for comparison. Densitometric analyses (see Materials and Methods) of similar gels containing known concentrations of purified SPN and IFS revealed a 1:1 molar stoichiometry of SPN to IFS in the complex.

The ability of IFS and SPN to form a complex was also assessed by size exclusion chromatography. Analysis of purified recombinant SPN (see Materials and Methods) by this method indicated an apparent molecular weight in solution of 52.4 ± 1.5 kDa (Figure 9), consistent with the predicted monomer size of the mature protein based on its primary sequence (48.4 kDa) and several previous reports indicating that SPN purified from streptococcal supernatant fluids is a monomer in solution [7,23,24]. However, a similar analysis of purified recombinant IFS (see Materials and Methods) indicated an apparent molecular weight of 31.5 ± 3.2 kDa (Figure 9), a value that was unexpected based on a molecular weight of 22.0 kDa predicted from the IFS-myc-HIS primary sequence. This suggests that IFS exists as a monomer with an extended shape or as an atypical dimer with Stoke's radius typical of a 31-kDa globular protein. Next, an assembled SPN-IFS complex was purified from the cytoplasm of recombinant *E. coli* (TOP10 strain harboring pMAM3.18) and subjected to chromatography with the resulting size of 67.6 ± 3.2 kDa (Figure 9) that was larger that either SPN or IFS alone. Importantly, this complex eluted in a resolving fraction that followed the void volume, which was calculated at 78.6 kDa (see Materials and Methods). Assembly of complex in vitro from purified IFS and SPN resulted in a major peak eluting in the void volume, indicating a possible aggregation event occurred in vitro (data curve not shown). The size of the more physiologically relevant in vivo assembled complex (67 kDa) indicates that one SPN molecule (48 kDa) interacts with one IFS molecule (22 kDa). This was confirmed by an SDS-PAGE analysis of several fractions obtained from the leading edge of the high molecular peak that revealed the presence of both SPN and IFS (Figure 10). Analysis of SDS-PAGE gels by densitometry with comparison to known concentrations of purified SPN and IFS indicated that the molar ratio of SPN to IFS was 1:1 in these complexes, in agreement with the gel filtration data as stated above.

### IFS Is a Competitive Inhibitor of SPN

**Figure 11:**
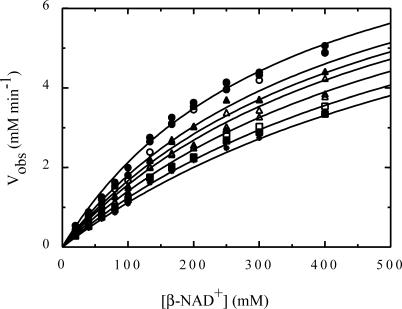
Kinetic Analysis of the Inhibition Mechanism of SPN by IFS The observed initial rate *(v_obs_)* of proteolysis of β-NAD^+^ by SPN (0.125 U/ml) was assessed in the absence (•) and presence of varying concentrations of IFS (○, 0.47 nM; ▴, 0.93 nM; Δ, 1.3 nM; □, 1.9 nM; ▪, 2.3 nM). The lines represent the nonlinear least-squares analysis of the competitive inhibition model with the parameters described in the text. Initial rates were measured, and the data were analyzed as described in Materials and Methods.

In order to determine the inhibition mechanism, the effects of IFS on the rate of hydrolysis of β-NAD^+^ by SPN were studied as a function of IFS concentration (Figure 11). The rate of β-NAD^+^ hydrolysis in the absence of IFS showed a hyperbolic increase with an apparent *K_m_* for β-NAD^+^ of 379 ± 74 μM and an observed *V_max_* of 9.9 ± 1.2 μM/min. Inhibition of SPN activity by IFS was determined by varying the concentration of IFS in the reactions. Increasing concentrations of IFS decreased the rate of β-NAD^+^ hydrolysis. Simultaneous fitting of these curves by a competitive model yielded a *K_m,app_* of 348 ± 54 μM for β-NAD^+^, a *V_max,obs_* of 9.6 ± 0.8 μM/min, and a *K_I,app_* of 2.0 ± 0.3 nM (Figure 11). The observed *K_m,app_* and *V_max,obs_* values were consistent to those obtained by fitting of the data by the Michaelis-Menten equation in the absence of any inhibitor. Simultaneous fitting of the curves by uncompetitive and noncompetitive models yielded parameters that fit the data poorly (fits not shown) and were inconsistent to the parameters obtained by fits of β-NAD^+^ hydrolysis in the absence of any inhibitor. The results demonstrate that SPN was inhibited by IFS in a competitive manner as indicated by the consistent Michaelis constants. The results also indicated that the complex between SPN and IFS was tight in comparison with the substrate β-NAD^+^.

## Discussion

In this study, we have described IFS, a novel endogenous inhibitor of SPN, the secreted NAD-glycohydrolase of *S. pyogenes*. IFS forms a stable complex with active SPN and functions as a competitive inhibitor of its β-NAD^+^ substrate. Furthermore, IFS proved to be essential for the ability of *S. pyogenes* to express SPN. As a practical consequence, co-expression of IFS and SPN proved to be a highly successful strategy for production of SPN in *E. coli*. To our knowledge, *ifs* represents the first molecularly characterized gene product encoding an endogenous inhibitor for a β-NAD^+^−consuming microbial toxin or for any NAD-glycohydrolase to date from either the prokaryotic or eukaryotic kingdoms.

In order to produce and secrete universally toxic molecules, bacterial pathogens have evolved several strategies to protect themselves from the self-toxicity of these molecules. For example, some toxins, like the calmodulin-dependent adenylate cyclases or the cholesterol-dependent cytolysins, require a co-factor found exclusively in a host compartment for activity (for reviews, see [25,26]). Other toxic enzymes, most notably broad-spectrum proteases, are secreted as inactive precursors whose conversion to an active form can be subsequently regulated both temporally and spatially [27]. A less common strategy involves the production of a cytoplasmic inhibitor. Although an inhibitor of the streptococcal SpeB cysteine protease was recently discovered [28], the best-studied example of this class is the staphostatin-staphopain system of the staphylococci [29]. Staphopains are secreted cysteine proteases that are specifically inhibited by the staphostatins, which are small (approximately 13 kDa) and acidic proteins that, similar to IFS, reside in the staphylococcal cytoplasm. Also like IFS, the staphostatins form a stable noncovalent complex at a 1:1 molar ratio with their cognate staphopain in their fully folded and active conformations [30]. Three-dimensional structures of the staphostatin-staphopain complex have revealed a competitive mechanism of inhibition whereby the staphostatin binds to the staphopain in a substrate-like manner forming a long-lived inhibitor-enzyme complex [30−34]. The observations that co-expression of a staphostatin increases the efficiency of staphopain production when expressed in *E. coli* [13] and that deletion of a staphostatin has a profound affect on the ability of the mutant to produce its cell wall and export proteins [35] have suggested that the function of the staphostatins is to prevent improper degradation of cytoplasmic proteins by premature activation of staphopains in the cytoplasmic compartment.

Similar to a staphostatin, the function of IFS may prevent the premature activation of the enzyme in the bacterial cytosolic compartment. However, while numerous inhibitors for various proteases have been described, including a recent discovery of an SpeB endogenous inhibitor [28], endogenous inhibitors of β-NAD^+^−consuming toxins are less common. Several prior reports have characterized endogenous NAD-glycohydrolase inhibitory activity in extracts of *Bacillus subtilis* and *Mycobacterium phlei* extracts [36,37]. However, identification of the genes that encode these activities has not been reported. The ability of IFS to bind in a tight complex with a 1:1 molar stoichiometry with SPN and to act as a competitive inhibitor of SPN's β-NAD^+^ substrate implies that IFS may function similarly to the staphostatins and form a complex that makes contact with residues at or near the catalytic center of SPN. Alternatively, IFS may act as an effective molecular mimic of β-NAD^+^ and function as a noncleavable substrate. Should the latter mechanism prove to be the case, IFS may act as a broad-spectrum inhibitor of β-NAD^+^−consuming toxins.

An important and unusual feature of both IFS and the staphostatins is that they are located in the cytoplasmic compartment, yet they bind and inhibit the activities of their cognate enzymes when these are in their active and fully folded conformations. While this property is valuable if the primary function of these proteins to inhibit the potentially toxic activities of the prematurely activated toxins, it also implies that these proteins become folded prior to their secretion. In this scenario, IFS may act to protect the cell from a small population of SPN molecules that drift off the Sec secretion pathway and then fold in the cytoplasmic compartment. This would explain why IFS is essential in both native and heterologous hosts that contain NAD-glycohydrolase−proficient SPN. This function would also be consistent with the apparent linkage between the NAD-glycohydrolase−deficient *spn* allele and the truncated allele for *ifs*. These strains produced an SPN polypeptide but lacked a cytoplasmic inhibitory activity. Thus, in the absence of enzymatic activity, an IFS-mediated inhibitory activity is not required, and this would likely reduce any selective pressure to maintain the full-length *ifs* allele. The loss of inhibitory activity does not rule out the possibility that IFS may have some other essential role. Multiple functions have been attributed to the chaperones of the type III secretion pathway, including secretion targeting of the effector-chaperone complex, unfolding cognate effectors, protection of effectors from proteases, and establishing a temporal hierarchy for effector secretion [38]. Many of these same functions are likely required for successful targeting for Sec pathway secretion and delivery of SPN to the CMT pathway. Thus, any of these functions may also be provided by IFS. However, it remains to be determined whether the truncated allele is expressed and whether it is required for expression of the NAD-glycohydrolase−deficient SPN or its translocation into the host cell cytosol via CMT.

The existence of an NAD-glycohydrolase−deficient allele of *spn* raises some interesting questions on the function of SPN and its repertory of enzymatic activities in pathogenesis. An important role is supported by the fact that several studies have shown that the gene for SPN is present in virtually all *S. pyogenes* isolates examined to date [1,39]. Two major alleles of *spn* were found, and there was an associated lack of immunoreactive protein for one allele [1]. We have shown here that the lack of detectable protein is directly caused by an SpeB-dependent degradation event through the use of a biochemical inhibitor and mutagenesis of *speB* (see Figure 5), and this observation was also noted in a recent study [20]. It is unclear if the variability in detectable SPN protein is due to an alteration in SpeB expression or whether SpeB is inefficient at degrading the active SPN allele. Furthermore, we have demonstrated an association of the *spn* allele exhibiting NAD-glycohydrolase activity with a full-length *ifs* allele. Due to the small sampling of strains used in this study, a more thorough study on the correlation of these alleles could be useful to establish whether the *ifs* nonsense mutation induces a resulting mutation in *spn* to prevent self-toxicity by β-NAD^+^ depletion. Nevertheless, the fact that the NAD-glycohydrolase−deficient allele is apparently widely distributed in the streptococcal population indicates it may have an as-yet-unidentified contribution to pathogenesis that is independent of an NAD-glycohydrolase activity. Thus, a more thorough understanding of SPN's activities and its contribution to virulence via the CMT pathway will require considerably more experimentation.

## Materials and Methods

### Bacterial strains and culture conditions.

Molecular cloning experiments utilized *E. coli* TOP10 (Invitrogen, Carlsbad, California, United States). The *S. pyogenes* strains JRS4, SLO1 (JRS4 SLO^−^), SPN1 (JRS4 SPN^−^), SF370, HSC5, JWR10 (HSC5 *speB_C192S_*), and MNN100 (HSC5 RopB^−^) have previously described [2,40−42]. Luria-Bertaini medium was used for culture of *E. coli,* while routine culture of *S. pyogenes* utilized Todd-Hewitt medium (BBL) supplemented with 0.2% yeast extract (Difco, BD Biosciences, San Diego, California, United States) (THY medium). For certain assays (see below), *S. pyogenes* was cultured in Dulbecco's modified Eagle media (without glucose, phenol red, or pyruvate) supplemented with 2.5% yeast extract and 2% glucose (DMEM-YE medium). Antibiotics were routinely used to maintain selection for plasmids and were added to media at the following concentrations: kanamycin, 50 μg/ml for *E. coli* and 500 μg/ml for *S. pyogenes;* erythromycin, 750 μg/ml for *E. coli* and 1 μg/ml for *S. pyogenes;* and ampicillin, 100 μg/ml for *E. coli*. Where indicated, the cysteine protease inhibitor E-64 (Sigma, St. Louis, Missouri, United States) was added to THY medium at a final concentration of 28 μM.

### DNA and computational techniques.

Plasmid DNA was isolated by standard techniques and used to transform chemically competent *E. coli* [43] and to transform *S. pyogenes* by electroporation [44]. Restriction endonucleases, ligases, and polymerases were used according to the manufacturers' recommendations. Chromosomal DNA was purified from *S. pyogenes* as previously described [44]. Fidelity of all DNA sequences generated by PCR was verified using fluorescently labeled dideoxynucleotides (Big Dye terminators; Applied Biosystems, Foster City, California, United States) and the appropriate oligonucleotide primers in DNA sequencing reactions according to the recommendations of the manufacturer. Identification of gram-positive secretion signal peptides utilized the SignalP model [45]. The alignment of *spy0166 (ifs)* open-reading frames was generated using the ClustalW algorithm [46].

### Cloning and expression of *spn* and *ifs.*

Plasmids for the cloning and expression of *spn* and *ifs* in *E. coli* were based on the pBAD/gIIIB expression vector (Invitrogen). Primers MAM98 and MAM84 (Table S1) were designed to amplify a single fragment containing *spn* and *ifs* from JRS4 chromosomal DNA so that the sequences encoding the signal sequence of *spn* were replaced with a sequence encoding a 6X-His affinity tag. The fragment was digested with NcoI and BstBI using sites embedded in the primers and inserted between the NcoI and BstBI sites of pBAD/gIIIB. In the resulting plasmid (pMAM3.14), *spn* was grafted in-frame with a gene III signal sequence followed by the 6X-His sequence and *ifs* was grafted in-frame with vector sequences specifying a C-terminal c-myc epitope tag followed by a 6X-His affinity tag. A derivative of pMAM3.14 was constructed with primers MAM101 and MAM102 (Table S1) using an “inside-out” PCR strategy [47] in order to remove the 6X-His segment introduced into *ifs*. The resulting plasmid (pMAM3.18) contains an additional PstI site introduced by the primers that was used to recircularize the PCR product. A similar strategy using primers MAM93B and MAM84 (Table S1) and a pMAM3.14 template was used to construct a plasmid (pMAM3.8) that lacked the segment encoding the 6X-His tag upstream of *spn*, while retaining the 6X-His tag sequence on *ifs*. Primers MAM103 and MAM84 (Table S1) were used to amplify and insert *ifs* between the NcoI and BstB1 sites of pBAD/gIIIB. The resulting plasmid (pMAM3.19) expressed IFS with C-terminal c-myc and 6X-His tags in the cytoplasmic compartment of its *E. coli* host. This plasmid was modified to remove the sequence encoding the 6X-His tag with primers MAM101 and MAM102 using an “inside-out” strategy as described above. The resulting plasmid (pMAM3.21) expresses IFS with a c-myc epitope tag.

### Construction of *ifs* in-frame deletion.

Primers MAM78B and MAM79B (Table S1) amplified a 1.4-kb fragment containing the entire JRS4 *ifs* open reading frame with flanking sequences in *spn* and *slo*. The fragment was inserted into a standard vector (pCRII; Invitrogen) using XhoI sites embedded in the primers. An “inside-out” PCR strategy using primers MAM80 and MAM81 (Table S1) introduced an in-frame deletion of 0.46 kb into the *ifs* open-reading frame. Sites for SalI (embedded in primers MAM80 and MAM81) were used to insert the fragment containing the deletion and flanking sequences into the shuttle vector pJRS233 [48]. The resulting plasmid (pMAM3.3) was then used in attempts to replace the wild-type *ifs* allele as described in the text according to a previously described method [42]. Wild-type and mutant alleles were distinguished by PCR amplification of chromosomal DNA using primers MAM79B and MAM87S (Table S1) that yielded products of 1,083 bp (wild-type) or 627 bp (*ifs* deletion), and assignment was confirmed by PCR using primers MAM110 and MAM112 (Table S1) that yielded a 480-bp product only in the presence of wild-type *ifs*.

### Ectopic expression of *ifs* in *S. pyogenes.*

Primers MAM108 and MAM112 (Table S1) were used to amplify *ifs* with its native ribosome-binding site from JRS4 chromosomal DNA with the addition of DNA encoding a C-terminal HA epitope tag. The resulting 0.51-kb fragment was inserted between the EcoRI and PstI of the *E. coli*/streptococcal shuttle vector pABG5 [49] using sites embedded in the primers. In the resulting plasmid (pMAM3.32), expression of the gene for the modified IFS (IFS-HA) is controlled by the *rofA* promoter [49]. Expression of IFS-HA following introduction of pMAM3.32 into *S. pyogenes* was confirmed in Western blot analysis of cell extracts (see below) using an anti-HA antiserum (Sigma).

### Assays for SPN.

Expression of SPN protein was evaluated by Western blot analyses of *S. pyogenes* culture supernatant fluids [2] or from *E. coli* periplasmic or cytoplasmic fractions [50] using an antiserum that cross-reacts with SPN (anti-SLO antibody, lot No. 7317011; Golden West Biologicals, Temecula, California, United States) [2]. The NAD-glycohydrolase activity of SPN was assessed by either endpoint titer or determination of units of activity (in picomoles of β-NAD^+^ cleaved per minute), both as previously described [2]. For the latter, more than 50 units represents the lower limit of detection of the assay.

### Assays for IFS.

Expression of SPN protein was evaluated by Western blot analyses of cytoplasmic extracts of *S. pyogenes* or from periplasmic or cytoplasmic fractions [50] of *E. coli* using antisera that recognize the appropriate HA (see above) or c-myc (Sigma) epitope tags. To isolate streptococcal cytoplasmic extracts, bacterial cells (washed twice and resuspended in 10 mM Tris [pH 8.0]) were lysed using a high-speed reciprocating shaking device (FP-120; Savant Instruments, Holbrook, New York, United States), and the extract was separated by centrifugation (15,000×g, 10 min, 4 °C). The success of streptococcal cell lysis was verified by visualization of cytoplasmic proteins by SDS-PAGE analysis and supported by the gain of inhibitory activity in the complemented *ifs^−^* mutant strain. Activity assays for the ability of IFS to inhibit NAD-glycohydrolase activity were as follows. For *E. coli* fractions, equal volumes of induced periplasm (0.05 μg total protein), cytoplasm (0.5 μg total protein), or buffer (1× PBS) were mixed and incubated with β-NAD^+^ (0.133 μM) for 0 to 50 min in a 96-well microtiter plate. For *S. pyogenes* fractions, cell-free overnight culture supernatants from JRS4 (diluted 1:15 in PBS) were mixed with streptococcal cytoplasmic extracts (50 μg total protein) or buffer (1× PBS) and incubated with β-NAD^+^ as described above. Units of NAD-glycohydrolase activity detectable at the end of the incubation period were then determined as described previously [2].

### Ni-NTA agarose pull-down assay.

The various *E. coli* strains described in the text were cultured under inducing conditions (see below), and cytoplasmic and periplasmic fractions were prepared [50]. For pull-down assays, periplasm (7.5 μg total protein), cytoplasm (75 μg total protein), or buffer (1× PBS) was coincubated for 15 min at room temperature prior to the addition of 50 μl of a 50% solution of Ni-NTA agarose (Qiagen, Valencia, California, United States). The mixtures were incubated for an additional 15 min at room temperature, and the agarose beads were recovered by centrifugation. Following aspiration of the supernatant fluids, the beads were washed three times in an equal volume of wash buffer (50 mM NaH_2_PO_4_, 300 mM NaCl, 20 mM imidazole [pH 8.0]), and bound proteins were eluted by incubation with an equal volume of elution buffer (50 mM NaH_2_PO_4_, 300 mM NaCl, 250 mM imidazole [pH 8.0]) for 15 min at room temperature. Beads were removed by centrifugation, and proteins in the supernatant fluids were subjected to Western blot analyses using antisera to detect SPN or IFS. Controls included extracts prepared from *E. coli* strains containing the vector alone (pBAD/gIIIB) or pBAD/gIII/calmodulin (pCALM), which expresses calmodulin with 6X-His and c-myc tags (Invitrogen).

### Purification of SPN.

TOP10 cells containing pMAM3.18 were induced at mid-log phase (OD_600_ 0.5) for 4 h using 0.2% l-arabinose (final concentration). Periplasm was isolated as previously described [50] and dialyzed against buffer composed of 50 mM phosphate buffer and 300 mM NaCl (pH 7.0). The sample was then subjected to metal-affinity chromatography over a TALON Superflow resin using the washing and elution conditions recommended by the manufacturer (BD Biosciences). Fractions containing SPN were dialyzed in a buffer containing 20 mM MES (pH 5.8) and subjected to chromatography over a Resource S ion-exchange column (Amersham Biosciences, Little Chalfont, United Kingdom) using a linear NaCl gradient (0 to 500 mM) to elute SPN. Fractions were analyzed by SDS-PAGE, and those fractions containing greater than 95% SPN were pooled (see Figure 10).

### Purification of IFS.

TOP10 cells containing pMAM3.19 were induced at mid-log phase (OD_600_ 0.5) for 4 h using 0.2% l-arabinose (final concentration). Cells were prepared by extraction of periplasm and then resuspended in TALON column buffer (300 mM NaCl, 50 mM Na-phosphate [pH 7.0]) and lysed by sonication. The lysate was then subjected to metal affinity chromatography over a TALON Superflow resin using conditions recommended by the manufacturer (BD Biosciences). Fractions containing IFS were dialyzed against buffer containing 20 mM Tris-HCl (pH 8.5) and subjected to chromatography over a Resource Q ion exchange column (Amersham Biosciences) using a linear NaCl gradient (0 to 500 mM) to elute IFS. Fractions were analyzed by SDS-PAGE, and those fractions containing greater than 95% SPN were pooled (see Figure 10).

### Molecular weight estimates.

Size exclusion chromatography was used to estimate the molecular weights of SPN, IFS, and the SPN-IFS complex. All chromatography was performed on a Superdex 75 HR 10/30 column (Amersham Biosciences) using a flow rate of 0.3 ml/min. Low-molecular-weight protein standards (Amersham Biosciences, see Figure 9) and test samples were equilibrated and developed in a buffer of 20 mM Tris (pH 8.5) plus 100 mM NaCl. Standards were analyzed (200 μl) on three separate occasions, and the average elution volumes were recorded. The value of *K_av_* was determined for each standard using the equation *K_av_* = (V_e_ − V_o_)/(V_t_ − V_o_), where V_e_ is the elution volume, V_o_ is the void volume (7.9 ml), and V_t_ is the bed volume of the column (24 ml). For each standard, *K_av_* was plotted against log M_r_ (molecular weight). Molecular weight estimates represented the average derived from at least three independent experiments.

### Stoichiometry of the SPN-IFS complex.

The protein concentration of the SPN-IFS complex isolated by size exclusion chromatography (see above) was determined using the bicinchoninic acid assay (Sigma) and a BSA protein standard (Sigma). Various concentrations of the complex were subjected to SDS-PAGE along with known concentrations of purified SPN and IFS. Gels were stained with Coomassie R-250, and an image captured on Kodak Image Station 2000MM was analyzed using Kodak 1D Image Analysis Software. Total pixel intensities of each band were obtained using the ROI (region of interest) function of the software. The intensities of bands from the known molar concentrations of SPN and IFS included on each gel were used to generate standard curves that were used to calculate the concentration of SPN and IFS from the pixel intensities of bands resolved from the complex. The calculated molar ratio reported was based on two independent experiments, each of which yielded an identical result.

### Kinetic analysis of the inhibitory mechanism of IFS.

The inhibition mechanism of IFS was determined in kinetic assays of SPN activity monitored by fluorescence detection of hydrolysis of β-NAD^+^ (Sigma), as previously described [8]. Briefly, JRS4 cells were grown overnight in DMEM-YE and centrifuged at 6,000 rpm for 10 min, and collected supernatants were sterile-filtered. One unit of SPN activity was defined as the activity in 1 ml of supernatant from the overnight growth. At least three preparations of SPN were used throughout these studies, and they yielded consistent and overlapping rates of hydrolysis. SPN (0.125 U/ml) was incubated with varying concentrations of β-NAD^+^ (up to 1.2 mM) in PBS at 37 °C for varying times (up to 50 min). Reactions were quenched by the addition of 5N NaOH, and the fluorescence of residual β-NAD^+^ was detected using excitation wavelength of 380 nm and an emission wavelength of 455 nm on a Perkin-Elmer LS55B using the plate reader accessory. The residual concentration of β-NAD^+^ was calculated as previously described [2]. Plots of the concentration of β-NAD^+^ hydrolysis with time were fit to a straight line to obtain the observed velocity *(v_obs_).* The rates of β-NAD^+^ hydrolysis as a function of β-NAD^+^ concentration were fit by the Michaelis-Menten equation to determine the apparent Michaelis constant *(K_m,app_)* and the maximum rate *(V_max,obs_)* [51]. The inhibition mechanism of IFS was determined in reactions of varying concentrations of purified IFS (up to 2.3 nM) with SPN (0.125 U/ml) and varying concentrations of β-NAD^+^ as described above. The observed velocities as a function of β-NAD^+^ and varying concentrations of IFS were simultaneously fit by competitive, noncompetitive, and uncompetitive inhibition models to determine the apparent inhibition constant *(K_I,app_),* the apparent Michaelis constant *(K_m,app_),* and the observed maximal velocity *(V_max,obs_)* [51]. The model that gave the best fit was used in determination of the inhibition mechanism. Nonlinear least-squares analysis was performed with Scientist (Micromath, Salt Lake City, Utah, United States). Errors in the reported parameters are ±2 SDs.

## Supporting Information

Table S1 Primers Used in This Study (31 KB PDF)

### Accession Numbers

The GenBank (http://www.ncbi.nlm.nih.gov/Genbank) accession numbers for the genes and gene products discussed in this paper are IFS (DQ093072), Spy_M30129 (NC_004070), Spy_M180164 (NC_003485), *spy0166* (NC_002737)*,* strain HSC5 (DQ093073), and strain JRS4 (DQ093072).

## Abbreviations

β-NAD^+^: β-nicotinamide adenine dinucleotide
CMT: cytolysin-mediated translocation
IFS: immunity factor for SPN
SPN: *Streptococcus pyogenes* NAD-glycohydrolase
