# Supplementary material for: A Novel Endogenous Inhibitor of the Secreted Streptococcal NAD-Glycohydrolase
Source: PLoS Pathog. 2005 Dec 2;1(4):e35. doi: 10.1371/journal.ppat.0010035 (PMC1298937; doi:10.1371/journal.ppat.0010035)
Supplement: Supplementary file 1 [file ppat.0010035.st001.pdf]

TABLE 1: Primers used in this study

Name	Sequence[a]
MAM78B	CCGCCGCTCGAGGTGACAAG TTTTGCTGAAGATATGGC
MAM79B	CCGCCGCTCGAGCAGCTTTCTTAACGCCTTCTTTAGG
MAM80	AAACGCGTCGACCACCTTATACATATTGTTACTTCC
MAM81	AAACGCGTCGACGAAACATTTTAGAATGATAAAAAGG
MAM84	TAGAAAGCTTCGAAAAAATGTTTCTATTGTTCTTTCGACC
MAM87S	GCTGTCTTAGCAGAGATTGATGCC
MAM93B	CATAGCACCATGGTTAGTGGCAAAGAAAACAAAAAAAGCG
MAM98	CATAGCACCATGGTACATCATCATCATCATCATGTTACCCGT GTTAGTGGCAAAGAAAACAAAAAAAGCG
MAM101	AAAAAACTGCAGGTTTAAACGGTCTCCAGCTTGGCTG
MAM102	AAAAAACTGCAGTCAGTCGACGGCGCTATTCAGATCCTC
MAM103	CATAGCACCATGGTATGATAACAGGAGGAATTAACCAT GTATAAGGTGCCAAAGGGTTTAG
MAM108	GACGCGGAATTCTAGCAAGATAGAATGCAAGATAGGAAG
MAM110	GACGCGGAATTCTAGATGTATAAGGTGCCAAAGGG
MAM112	AAAAAACTGCAGTCAAGCATAATCTGGAACATCATATG GATAAAATGTTTCTATTGTTCTTTCGACC

[a]Underlined sequences denote restriction sites incorporated into primer.
